# Cheaper Medicines for the Better Off? A Comparison of Medicine Prices and Client Socioeconomic Status Between Chain and Independent Retail Pharmacies in Urban India

**DOI:** 10.34172/ijhpm.2020.214

**Published:** 2020-11-09

**Authors:** Rosalind Miller, Catherine Goodman

**Affiliations:** Department of Global Health and Development, Faculty of Public Health and Policy, London School of Hygiene and Tropical Medicine, London, UK.

**Keywords:** Private Sector, Pharmacies, Socioeconomic Status, LMICs, India

## Abstract

**Background: **The growth of chain pharmacies in India, and other low- and middle-income countries (LMICs), is challenging the status quo of pharmacy retail markets which have historically been dominated by independent pharmacies. This raises the question of whether such organisations will have a positive impact on affordability and access to medicines.

**Methods:** This paper draws on a standardised patient (SP) survey to measure the prices of medicines and expenditure on consultations for two tracer conditions (suspected tuberculosis [TB] in an adult and diarrhoea in an absent child) at a random sample of 230 chain and independent pharmacies in Bengaluru. Asset data were collected from 808 exit interviews with pharmacy customers to determine socioeconomic profiles of clients.

** Results:** Chain pharmacies were found to provide lower priced medicines for patients seeking care for diarrhoea and TB, with expenditure also lower for diarrhoea patients, compared to independent pharmacies. This was seemingly driven by lower prices rather than number of medicines dispensed or prescribing habits. Despite the availability of cheaper medicines, chains served wealthier clients, compared to independent pharmacies.

**Conclusion:** The findings indicate the potential for chains to contribute to improving medicine affordability as they expand. However, any attempt to leverage this organisational model for public health good would need to take account of the current client-mix of these pharmacies and be accompanied by appropriate regulatory constraints in order to realise the potential benefits for poorer groups.

## Background

**Key Messages**

** Implications for policy makers**

- Chain pharmacies in Bengaluru, India, were found to provide lower priced medicines compared to independent pharmacies for patients seeking care for diarrhoea and tuberculosis (TB).
- Chain pharmacies have the potential to contribute to improving medicine affordability as they expand.
- Chains are currently patronised by relatively wealthy clients, indicating that such cost-reductions are currently disproportionately benefiting higher socioeconomic status (SES) groups.
- Any attempt to leverage the chain pharmacy model for public health good should be accompanied by appropriate regulatory constraints to ensure benefits are extended to poorer groups.

** Implications for the public**

Access to affordable medicines is a key concern in many low- and middle-income countries (LMICs). This study, conducted in urban India, found that chain retail pharmacy medicine prices were lower relative to medicines sold in independent pharmacy prices for two conditions. However, the customers using chain pharmacies and benefitting from these price reductions are currently relatively wealthier than those using independent pharmacies. Despite the high-end look and feel of chain pharmacies, potential customers (especially poorer groups) should be aware of these price differences.

Retail pharmacies represent an important component of the health system in low- and middle-income countries (LMICs). Owing to their geographical accessibility, long opening hours, and availability of medicines they are often patients’ first port of call when illness arises.^1^ Pharmacy retail markets in LMICs have traditionally been dominated by local, independently owned pharmacies.^2,3^ These pharmacies are widely used but their practice has been judged to be poor. Insufficient history taking, a lack of adherence to treatment guidelines, and inappropriate dispensing of medicines are commonplace.^1,4,5^ Moreover, up to 90% of the population in LMICs purchase medicines through out-of-pocket payments, with medicines accounting for the largest family expenditure item after food.^6^ As a result, medicines are unaffordable for many.

 As the retail pharmacy market has matured, the entry of chain pharmacies has been noted in Africa, Asia and South America, with a particular presence in countries such as India, The Philippines, Mexico, Brazil, Chile, South Africa, Nigeria, Kenya, and Uganda.^2,3,7^ This has led to emerging interest amongst the global health community in the potential for chains to improve pharmacy services. In particular, there are reasons to believe that chains have the potential to reduce prices in comparison to independent pharmacies, making them a welcome addition from an access to medicines perspective. Chains may be able to take advantage of scale economies to invest in cost-reducing and quality-enhancing technologies.^8^ They may buy directly from manufacturers, thus by-passing the serial mark-ups associated with a complex supply chain. Additional savings could be made if chains became large enough to experience buying power in the market. In high-income countries, experience has shown that pharmacy chains pass these cost savings on to customers and many compete on the basis of price.^9-11^

 Moreover, cost to patients depends on both price and product-mix dispensed. If chain staff receive fixed salaries and therefore face lower-powered incentives than those of independent pharmacy owners, they may be less likely to encourage poly-pharmacy and purchase of more expensive products and brands.^12^ In contrast, the profit-related incentives of independent pharmacy owners could lead to unnecessary, and sometimes inappropriate, dispensing.^4^

 However, chain employees may also face high-powered incentives if they are heavily rewarded for achieving sales targets.^13^ It is also possible that, rather than passing on any cost savings to patients in the form of cheaper medicines, chain pharmacies may use their greater brand power to raise prices. In addition, in many settings, chains may operate in relatively affluent neighbourhoods,^8^ and provide relatively high-end services, with better infrastructure, more qualified staff and additional facilities, which could feed through into higher drug prices.

 To date, research on price and expenditure in this evolving segment of LMIC pharmacy markets has been very limited. Only one study by Bennet and Yin was identified, which investigated the effect of chain entry on quality and prices of medicines in local markets in Hyderabad, India. They worked with a leading Indian pharmacy chain, MedPlus, using standardised patients (SPs) to collect data on medicine price and chemical quality of two antibiotics in independent pharmacies in markets with and without a MedPlus entrant, before and after chain entry. The resulting effect of chain entry on the market (compared with control markets) was a relative 5% improvement in drug quality and a 2% decrease in prices at existing independent retailers.^7^

 This paper aims to build on this limited evidence base by exploring prices of medicines and expenditure for two conditions – diarrhoea in a child and tuberculosis (TB) in an adult – utilising SPs at a random sample of chain and independent pharmacies in Bengaluru, India. To further explore the consequences of chain pharmacies for medicine access, we also compare the socioeconomic status (SES) of patients visiting chain and independent stores, based on exit interviews with real clients.

## Methods

###  Study Setting

 Chains account for around 4% of India’s 800 000 pharmacies but their rate of growth is rapid.^3^ The chains are concentrated in India’s big cities, with a larger presence in the South. This guided the choice of Bengaluru, the capital of the Southern Indian state of Karnataka and India’s third most populous city, as the site for this work. Bengaluru has a population of around 10 million, and despite its relatively favourable human development indicator score (0.753) and literacy rate (88.7%), income disparities are stark. On the one hand, the city is known as ‘The Silicon Valley of India’ with a booming, international IT industry and a growing middle class; yet on the other, it is home to 597 of Karnataka’s 2804 identified slum communities, where many inhabitants live below the poverty line.^14^ The average daily wage for salaried, urban workers in Karnataka was reported to be 519 Rs ($7.05) and 392 Rs ($5.33) in 2011-2012 for men and women, respectively.^15^ The median income of inhabitants living in one of Bengaluru’s slums is reportedly $1.5 (US) per day.^16^ Out of pocket expenditure accounts for over 70% of healthcare spending in India, indicating that most patients pay for their own medicines.^17^ Health-related costs have been shown to push Bengaluru residents into poverty.^16^

 At the time of data collection (November 2014-June 2015) there were 13 chains operating in the city, accounting for 529 (9%) of Bengaluru’s 5664 registered retail pharmacies.^18^ Chain size varied from two to over two hundred outlets and five chains also owned pharmacies outside of Karnataka. There are notable physical differences between the two pharmacy types: independent stores tended to have an open, on-the-street shop frontage, whereas most chains were enclosed, with air conditioning, and appeared neatly organised. Both chains and independent pharmacies stock a mix of innovator brands (brands under patent); ‘branded’ generics (products produced by Indian manufacturers, who advertise their products heavily in order to establish a reputation and improve market power^8^); and less commonly, non-branded generic products labelled only by the active ingredient. In India a distinction is made between ‘national’ and ‘local’ medicine manufacturers. National manufacturers tend to comply with Indian and international quality standards, whereas local manufacturers (who often produce non-branded generics) are difficult to regulate due to their vast numbers. Retail pharmacies stock a high number of nationally and locally produced products for common medicines in order to accommodate a wide range of doctor and customer preferences. Each manufacturer dictates a maximum retail price (MRP) for their product but the MRP for a particular compound can vary across brands. There is therefore some scope for price variation through brand substitution. The National Pharmaceutical Pricing Authority, an independent body, also stipulates a ceiling price for some essential medicines.^19^

###  Study Design and Data

####  Standardised Patient Survey

 Price data were collected by means of an SP survey. SPs are healthy, undercover fieldworkers trained to present with specific symptoms and history, and to record the care they receive.^20^ This method has been increasingly used to measure quality of care in pharmacy settings over the past decade.^21^ It is widely considered the gold standard in terms of quality of care measurement; the standardised presentation allows for direct comparison between providers, and it is free from the Hawthorne effect.^20^ SP data were utilised to compare outcomes in chain versus independent pharmacies, including deviation of medicine prices from the MRP, expenditure on the diarrhoea and TB case, and number of medicines dispensed.

 A stratified sample of chain (103) and independent (230) pharmacies were chosen, at random, from the Karnataka State Drug Control Department’s list of licensed pharmacies in Bengaluru. A small census verified that this list provided a comprehensive sampling frame. Sample size calculations were based on differences in quality measurements between pharmacy types (see Miller and Goodman^18^ for more details). The sample included eight of Bengaluru’s 13 chains – the largest seven and one of the six chains with 2-5 outlets. Six research assistants were trained to visit pharmacies and present standardised cases of diarrhoea in an (absent) child and suspected TB in the presenting adult. A substantial component of the training involved role play to practice both the presentation of the cases and personal back stories. SPs completed a number of pilots under the supervision of a senior research assistant, until we were confident that the presentations and associated dialogue would be perceived as authentic. The scenarios were presented in Kannada (the local language) and it was discussed how researchers should dress to ensure they appeared similar to a typical customer. At each pharmacy the SPs purchased any medicines that were recommended during the interaction and recorded the price of each individual medicine sold in a debrief questionnaire, completed immediately after each visit.

####  Exit Interviews With Patients 

 We conducted exit interviews with real customers at the same random sample of pharmacies used for the SP survey. During a second visit to the pharmacies, researchers approached each customer that left the pharmacy and, if they agreed to take part, administered the exit interview to up to 3 customers per outlet, covering personal characteristics, reasons for visiting the pharmacy, and ownership of a number of assets. A total of 808 exit interviews were completed^[1]^, deriving from 103 chain outlets and 166 independent outlets.

 We utilised an asset-based approach to measure and compare the wealth of pharmacy customers visiting chain and independent pharmacies.^22^ We collected data on a subset of assets contained within the national District Level Health Survey (DLHS) (2012-2013), selecting assets relevant to the Bengaluru city context^[2]^. For example, we did not ask about ownership of a tractor or cart driven by an animal as these are more applicable for rural areas. Others have shown this to be a valid approach in both India and elsewhere.^23,24^ We were then able to compare the wealth of our patient sample with that of the general population residing in the same district (Bengaluru urban district, Karnataka).

###  Analysis

####  Standardised Patient Survey 

 Analysis of price data was conducted only on pharmacies who sold medicines (some SPs were referred to a physician without being sold any medicines). Data from the childhood diarrhoea and TB case were analysed separately using Stata 15. To examine prices of medicines sold at chain and independent pharmacies in a standardised way, across different medicine types, we calculated the difference between the actual price paid for medicines by SPs and the MRP, presenting both mean (accompanied by standard deviations and *t* test) and median price differences, interquartile range, and *P* value from the Wilcoxon rank sum test of the hypothesis of equal medians in chains and independents. We also calculated expenditure per encounter and used regression analysis to explore the effect of outlet type on expenditure.

 To explore the effect of product mix on expenditure we conducted a regression analysis to assess the relationship between expenditure for the diarrhoea case and sale of prescription only medicines (POMs) in general, and more specifically, antibiotics, the most commonly dispensed POM type. Prior work had revealed that chains dispensed fewer POMs and fewer antibiotics for diarrhoea.^18^

####  Exit Interviews

 We derived our SES indices using principal components analysis (PCA) in Stata 15. Using the DLHS data only, we generated a wealth asset score using PCA applied to the subset of assets measured in both the DLHS and the patient exit survey. We then generated a wealth asset score in the patient exit data by applying the PCA weights from the aforementioned data.^22^ We then split the DLHS sample into wealth quintiles, as described by Filmer and Pritchett^25^ and applied the same asset score quintile cut-offs to the patient exit sample. We compared the relative economic status of clients patronising chains and independents, as well as comparing our sample to the general population of Bengaluru.

 In order to investigate whether these apparent wealth disparities between chain and independent customers were driven by the location of chains (potentially in wealthier areas), we conducted additional analysis on a subsample of pharmacies to compare the wealth of clients of chain and independent pharmacies that are in close proximity (on the basis that the SES of the catchment population is likely to be similar). For this purpose, we utilised ArcGIS to plot the Global Positioning System (GPS) locations of sample pharmacies. We then excluded chain pharmacies without an independent within 1km, and vice versa, and repeated the same analyses.

## Results

 Of the 333 shops visited by SPs, 67/103 chains (65%) and 151/230 independents (66%) sold medicine(s) to the patient with suspected TB. For the diarrhoea patient, these figures were 63/103 (61%) and (153/230) 67% for chains and independents, respectively.

###  Price

 Individual medicines for diarrhoea were sold at a price below the MRP at both chains and independents, but the discount was larger for chains (0.9 rupees versus 0.15 rupees, *P*= .005) (Table 1). Whilst 0.9 Rupees (US$ 0.014) appears to be a small discount, the mean price difference in chains expressed as a percentage of the MRP was 6%. The median price difference for both chains and independents was zero. For medicines sold for TB symptoms, there was weak evidence that chains had lower prices relative to the MRP (discount of 0.03 rupees in chains compared with 1.44 rupees over the MRP in independents, *P*= .097). The median difference between price paid for TB medicines and MRP was 0.2 rupees in chains and 0 in independents (*P*= .059).

**Table 1:** Price Difference of Medicines Sold to SPs From the MRP, Number of Medicines Received, and Expenditure Per Encounter (Rupees) for Chain and Independent Pharmacies (Rupees)

	**Diarrhoea Case**			**TB Case**		
	**Chains**	**Independents**	***P*** ** Value**	**Chains**	**Independents**	***P*** ** Value**
Mean price difference (SD)	-0.90 (2.34)	-0.15 (1.71)	.005	0.03 (6.04)	1.44 (6.67)	.097
Median price difference (IQR)	0 (-1.87 to 0.46)	0 (0 to 0.33)	.316	0 (-0.87 to 0.60)	0.2 (0 to 1.12)	.059
Mean number of medicines received	1.21	1.22	.886	1.37	1.51	.171
Mean expenditure (SD)	27.5 (18.3)	35.6 (22.1)	.011	46.4 (26.8)	42.7 (38.5)	.473
Median expenditure (IQR)	19 (13 to 37)	38 (18 to 47)	.002	49.5 (20 to 64)	29 (11 to 64)	.226

Abbreviations: SPs, standardised patients; MRP, maximum retail price; TB, tuberculosis SD, standard deviation; IQR, interquartile range. Price difference = price paid - MRP.

###  Expenditure

 In terms of expenditure by the SP, diarrhoea encounters at chains had a mean cost of 27.5 rupees (median 19) (Table 1). These encounters were significantly more expensive at independent pharmacies with a mean cost of 35.6 rupees and median of 38 (*P*= .011 and *P*= .002, respectively). There was no evidence of a difference between chains and independents in terms of expenditure for the TB encounter. Expenditure per condition is a function of the prices of the medicines sold, the mean number of medicines sold, and the product mix. The number of medicines sold did not differ at chain and independent pharmacies (1.2 at both outlet types for diarrhoea).

 The effect of product mix on expenditure is explored in Table 2. Model 1 shows that chains were 23% cheaper^[3]^ than independent pharmacies, after controlling for SP fixed effects (*P*= .014). Model 2 explores the effect of number of medicines sold; the chain dummy is unchanged in this model indicating price differences are not explained by differences in dispensed medicines. Model 3 shows that whether a POM was sold had little effect on the coefficient on the chain dummy, while model 4 shows that controlling for whether an antibiotic was sold reduced the coefficient on the chain dummy, but did not eliminate it, indicating antibiotic purchase may explain part but not all of the observed price difference.

**Table 2:** Effect of Pharmacy Type on Price: Diarrhoea Encounter

**Dependent Variable**	**ln (Price)**			
	**Model 1**	**Model 2**	**Model 3**	**Model 4**
Chain pharmacy	-0.258^a^ (0.104)	-0.259^b^ (0.997)	-0.249^a^ (0.094)	-0.229^a^ (0.968)
SP fixed effects	Yes	Yes	Yes	Yes
Number of medicines	-	0.448^b^ (0.105)	0.438^b^ (0.106)	0.427^b^ (0.102)
Prescription only	-	-	0.083 (0.103)	-
Antibiotics	-	-	-	0.356^b^ (0.091)
Observations	216	216	216	216
R2	0.04	0.12	0.12	0.15

Abbreviation: SP, standardised patient. Standard errors in parentheses. ^a^ *P* <.050, ^b^*P* <.010.

###  Socioeconomic Profile of Clients

 Figure shows the distribution across wealth quintiles of customers visiting chain and independent shops in our sample, compared to that of the general population. The figure shows a clear pattern of higher wealth of independent customers compared to the general population. Chain customers were found to be relatively wealthy compared to both the general population and independent customers. The clearest difference can be seen in the highest wealth category (quintile 5) which comprises 20% of the general population, compared to nearly half (45%) of independent customers and the majority (67%) of chain customers. Chi^2^ tests showed that differences in Q5 between the three groups were significant (*P*< .001 for all tests).

**Figure:**
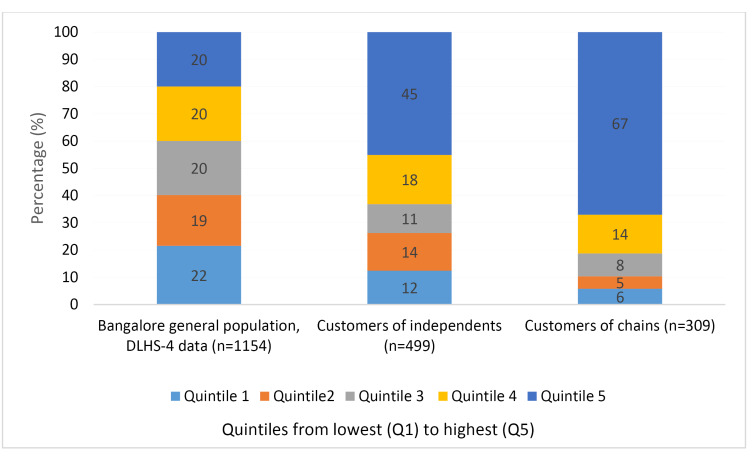
Wealth of Customers Patronising Chain and Independent Pharmacies Relative to the General Population of Bengaluru. Abbreviation: DLHS, District Level Health Survey.

 We repeated the analyses on a geographically matched dataset (not shown), which included 264 exit interviews from 90 independents and 237 exit interviews from 79 chains. The results were very similar to our original results in terms of customer wealth. The percentage of customers in the top quintile increased by 1 percentage point to 46% for independents and decreased to 66% (1% point) for chains, with the difference between the two, remaining significant (*P*< .001).

## Discussion

 This work used SPs to assess the actual prices of medicines sold in a large, representative sample of pharmacies in a major urban centre. We showed that prices of medicines sold at chain pharmacies were cheaper compared to those at independent pharmacies. Chains were undercutting the MRP more substantially than independents for the diarrhoea case. For the TB case, chain medicines followed the MRP and independents were charging more than the MRP (this difference was significant at the 10% level). In terms of expenditure by SP case, the diarrhoea encounter was significantly cheaper at chains and no difference was found for the TB case. Even after controlling for SP fixed effects and number of medicines sold, regression analyses showed that the diarrhoea encounter was 23% cheaper in chains, compared to independents (*P*< .010). This difference appears to be driven mainly by chains undercutting the MRP significantly more than independent pharmacies, although the sale of fewer antibiotics may also be a contributing factor. It is important to note that in absolute terms, chains were found to undercut the MRP by a very small amount (0.9 Rupees on average). When expressed as a percentage of the MRP, the mean price difference was -6%.

 There are two reasons why our price data may actually underestimate the price-cutting impact of chains. Firstly, most chains offer up to 10% discount on medicines to repeat customers which is not entirely captured by our SP patients who are unknown, first-time visitors to the pharmacy. Secondly, there are possible price spill over effects to independents, who may have decreased their prices in response to price competition from chains. Bennet and Yin^8^ showed that after the entry of a chain into a local retail pharmacy market, the price at independent shops fell by a non-significant 2%-4%. However, for non-national brands alone they found a significant 12%-15% price reduction. Similar spillover effects follwing the entry of low-cost chains have also been observed in other industries.^26,27^ The 6% reduction in MRP observed at chain pharmacies combined with 10% discounts for regular customers would result in meaningful medicine price reductions for many. Discussions with local chain executives revealed that these price reductions are possible due to exploitation of scale economies and the operational efficiency of the chain model. Bulk purchasing reportedly enables larger chains to enjoy purchasing discounts of around 3 to 7%. Further, their procurement processes usually bypass at least one layer of the supply chain. The use of technology in high-income country chains has been associated with increasing operational speed.^28^

 Customers shopping at chain pharmacies were better-off relative to those patronising independents. These results held after restricting the analysis to chain and independent pharmacies situated within 1km of each other, indicating that this finding was not purely driven by chain location. This may reflect the common practice by independent pharmacies of offering credit to those who cannot afford to buy medicines (which was reported in accompanying qualitative work). Additionally, the chains in Bangalore generally had a more high-end feel in terms of appearance, meaning that they may have been off-putting or intimidating to lower income groups. It is possible that the socio-economic mix of chain clients will change as the market evolves. Other industries have seen diffusion of retail chains in LMICs from big to smaller cities, and from upper to middle to poorer classes,^29^ meaning that we may see more lower-income chain patients over time.

 The higher SES of chain clients suggests that the relatively wealthy may be more sensitive to quality. This raises the question of whether wealthier clients are also getting better quality of care by patronising chains? Evidence on this is limited and inconclusive. Accompanying results from this project found no difference in quality of care in terms of case management, though chain pharmacies did sell fewer prescription only and harmful medicines to the diarrhoea SP but not to the TB one.^18^ Bennet and Yin found that medicines sold in chains were of similar pharmacopeial quality to those in independents.^7^ It is also feasible that customers perceive that a chain with better infrastructure will offer better quality medicines.

 It is also noteworthy that the profile of independent customers in our sample was considerably wealthier than that of the general population, indicating that the poorest members of the population were under-represented in both pharmacy types. This may reflect the high use of “informal providers” by poorer groups in India, who treat common illnesses with allopathic medicines, but have no government-recognised medical degree, or legal regulatory status.^30-32^

 The above results are drawn from looking at prices for only two conditions and would not be generalisable to outside urban areas, where chains are still very rare. However, they are likely relevant in the context of other Indian cities because, although different chains may predominate in these settings, there is no indication of major variation in business models.^13^ Evidence on chain pharmacies from other LMICs, in terms of price, SES of customers, and quality is lacking. In China, chain pharmacies reportedly have more stringent recruitment processes and training systems in place than independents.^33^ A nationwide survey reported that pharmacists working in chains had a better understanding of the concept of ‘pharmaceutical care.’ Further a higher percentage of chain pharmacists agreed that it was their duty to carry out such care and engage in practices such as provision of counselling to accompany the sale of medicines, monitoring for adverse drug reactions, and promoting drug safety. In Indonesia and Malaysia, chains are thought to provide better services than their independent counterparts, although there is little empirical evidence to support these claims.^34,35^ We did not identify any other studies from LMICs on relative price of medicines or SES of customers at chains and independents.

 Turning to the future for chain pharmacies, it is likely that, due to their ability to provide medicines at a lower price compared to independents, they will continue to expand their presence across India. Given that higher SES clients are currently the main benefactors of these lower prices, a set of requirements may be necessary to ensure that chain expansion includes presence in lower-income areas. If policy makers were interested in encouraging the development of chains, there are a number of regulatory or fiscal levers that could be set in motion to allow for this. These include tax breaks, relaxing licensing procedures, or liberalisation of foreign direct investment (FDI) for multi-brand retail trading (into which category the selling of pharmaceuticals falls). In 2012 the Government permitted FDI up to 51% of the equity of Indian entities engaged in such retailing.^36^ However, these investors are subjected to a host of conditions. In 2018 the Union Cabinet approved 100% FDI in single-brand retail but such relaxation has not yet been extended to multi-brand retail.^37^ However, based on current evidence the justification to utilise such levers to encourage the growth of chains on public health grounds in India remains weak.

## Conclusion

 In urban Bengaluru chain pharmacies were found to provide lower priced medicines for patients seeking care for diarrhoea and TB, with expenditure also lower for diarrhoea patients, compared to independent pharmacies. This indicates the potential for chains to contribute to improving medicine affordability as they expand, likely reflecting scale economies, more direct supply chains and greater purchasing power. However, despite these lower prices, chains were patronised by relatively wealthy clients, indicating that such cost-reductions are currently disproportionately benefiting higher SES groups. Any future attempt to leverage this organisational model for public health good would need to take account of the current client-mix of these pharmacies, and be accompanied by appropriate regulatory constraints in order to realise the potential benefits for poorer groups.

## Acknowledgements

 We would like to thank the Society for Community Health Awareness Research and Action, Bengaluru, in particular Dr. Thelma Narayan, for providing office space, support and guidance during the data collection process. We are grateful to Dr. Timothy Powell-Jackson for providing advice and commenting on an earlier draft of this manuscript.

## Ethical issues

 Ethical approval was obtained from The London School of Hygiene and Tropical Medicine Ethics Committee, London, UK, and the Society of Community Health Awareness Research and Action Institutional Scientific and Ethics Committee in Bengaluru, India. We obtained written consent for the exit interviews and received a consent waiver for the SP study. This approach has been used in a number of SP studies where the services being researched are accessible to the general public and data collection poses minimal risk to providers.^20^

## Competing interests

 Authors declare that they have no competing interests.

## Authors’ contributions

 RM and CG designed the study; RM oversaw data collection and analysed the data, CG contributed to data interpretation; RM drafted manuscript and CG provided critical review and comments.

## Funding

 This work was supported by an Economic and Social Research Council (ESRC) PhD studentship and an ESRC post-doctoral fellowship [grant number ES/T006854/1].

## Endnotes

 [1] Clients who refused to participate in exit interviews were not recorded, so it was not possible to calculate a response rate. [2] Refrigerator, telephone (landline), mobile phone, sewing machine, tap/running water, radio, scooter/motorcycle, washing machine, car/jeep/van, watch/clock, computer without internet, computer with internet, cooler/AC, number of bedrooms. [3] (e ^-0.2578^ – 1)*100 = -22.7.
